# Using an AI-Nurse to Help Teach Graduating Medical Students to Take Overnight Call

**DOI:** 10.1007/s40670-025-02335-6

**Published:** 2025-02-24

**Authors:** Christina Weaver, Nicholas J. Caputo, Connor Yost, Scott A. Hauert

**Affiliations:** https://ror.org/05hr6q169 grid.251612.3 0000 0004 0383 094X A.T. Still University School of Osteopathic Medicine in Arizona, E. Still Circle, Mesa, AZ 5850 USA

**Keywords:** Artificial intelligence, AI, Chatbot, UME-GME transition, Simulation, Residency readiness

## Abstract

Artificial intelligence (AI) can help prepare graduating medical students for responding to nursing texts, something few interns are prepared to do on day 1 of residency. An AI-nurse could enhance that preparation by paging and corresponding with fourth-year medical students, requesting urgent orders during an overnight on-call simulation.

Graduating medical students are typically not well-prepared to cross-cover patients or answer urgent nursing pages on their first day of residency. This gap in the transition from UME-GME has been called out in the education literature with various forms of simulation-based education aiming to bridge it; however, scalable solutions have proven elusive [1, 2].

To create higher fidelity, more robust simulations to address these gaps, researchers are increasingly turning to AI. For example, intelligent tutors provide personalized feedback while synthetic patients simulate difficult conversations to address critical training issues for medical students [3, 4]. Similarly, open-source medical information platforms reduce mountains of raw data into usable information that support evidence-based clinical decision-making. These applications illustrate how AI in general, and large language models in particular, are being used to improve medical education and practice.

Our objective was to contribute to the field by developing an easy-to-implement, cost-effective, high-fidelity method of simulating nurse pages to help advance the “on-call” clinical reasoning skills of fourth-year medical students enrolled in a residency readiness program. To achieve this, we created “Althea,” an AI-nurse chatbot that pages “on-call” medical students, requesting urgent orders for a simulated patient.

Using a framework for developing chatbots, we worked through five stages to train our AI-nurse. The first stage identified its role, which defined its persona and function (i.e., nurse). This influenced the type of responses it provided and the tone it used. The second stage established the chatbot’s objective. In this case, the objective was to respond to on-call–related questions from the student. The third stage refined the process of conversation by categorizing the prompts and optimizing them to ensure a logical flow and continuity. The fourth stage consisted of drafting prompts to reflect the chatbot’s tone, language, and personality to make its interaction suitable for the target audience of medical students. In the fifth stage, we created the database of domain knowledge that allowed Althea to provide accurate, context-appropriate responses.

The domain knowledge used to create the different scenarios was developed by this paper’s second author and the responses generated by Althea were reviewed by the third author. In each case, the responses were correct. In those instances where Althea could not provide an answer supported by its existing knowledge base, it responded with the equivalent of “I don’t know” rather than generate or “hallucinate” an inaccurate response.

Although conversational via text, Althea’s responses include *only* clinical data requested by the student, such as updated vital signs, patient appearance, current medications, and recent studies. In this case, AI-nurse pages were automatically generated during evening hours, simulating the experience of being on-call overnight and the urgency of communication among the hospital team, while challenging the students’ clinical reasoning skills when responding to unfamiliar, potentially stressful conditions. To the best of our knowledge, this is a novel application of AI in medical education.

In April 2024, 150 fourth-year osteopathic medical students participated in the Residency Readiness program as part of their required curriculum. That cohort was split into four groups of approximately 30–40 students per group, with each group participating in the same activities during different weeks of a 1-month period. To prevent other students from hearing about the cases and to prevent the sharing of information, only the 30 students in the final group were selected to receive the AI-nurse texts.

After 4 months of residency, the 30 participants that received the AI-nurse texts were asked to complete an anonymous, five-question survey about the AI-nurse experience. The survey focused on four key dimensions: overall experience, realism, fluidity, and any problems encountered during the AI-nurse interactions. The existing Residency Readiness project and amendment to add to this survey were deemed minimal risk/exempt by the A.T. Still University, Arizona IRB.

Seventeen of the 30 (57%) participants in the AI-nurse paging simulation responded to the post-intervention survey with Likert scale question-specific equivalents of 1 = very poor, 2 = poor, 3 = neutral, 4 = good, and 5 = very good.

Most students reported an overall positive experience with 10/17 (58.8%) finding it “Very positive,” and 2/17 (11.8%) finding it “Somewhat positive.” Two of 17 students (11.8%) were “Neutral,” 2/17 (11.8%) selected “Somewhat negative,” and 1/17 (5.9%) reported a “Very negative” experience.

The realism of the AI-nurse interactions was generally rated highly, with an average score of 4.24/5.00 and no rating lower than 3, indicating a strong sense of authenticity in the AI’s responses.

Fluidity, in terms of smoothness and naturalness of the AI-nurse interaction, also received favorable ratings, with an average score of 4.18/5.00 and most respondents rating fluidity a 4 or 5.

Only one student (5.9%) reported a problem during the interaction, stating “I asked a clarifying question, and the nurse never got back to me.” The remaining 16 students (94.1%) did not report any problems.

Results of this preliminary study indicate the AI-nurse provided a generally positive and realistic learning experience for most students, with comments such as “super cool” and “great training.” High ratings for both realism and fluidity coupled with the low incidence of problems encountered suggest that the AI-nurse was effective in simulating realistic nurse-to-doctor communications.

Although promising, there are some important limitations to the results. The survey instrument was short and designed to gather basic functionality and user experience information and thus has not yet been validated. Additionally, the sample size was small, warranting some caution about generalizing the findings. Because the survey occurred 4 months after the event, there is an increased risk of recall bias among the respondents.

Future studies with larger sample sizes and additional qualitative feedback could provide deeper insight to those aspects of the AI interaction students find most beneficial or problematic. A more robust evaluation of individual student and AI-nurse conversations is warranted to assess student communication and clinical decision-making skills during each simulated on-call interaction. Our data suggests that with further refinement, the AI-nurse paging program could be an easily scalable and highly effective educational tool for medical students in the transition to residency.

## References

[1] Hauer KE, Williams PM, Byerley JS, Swails JL, Barone MA. Blue skies with clouds: envisioning the future ideal state and identifying ongoing tensions in the UME–GME Transition. Acad Med. 2023. 10.1097/ACM.0000000000004920. 10.1097/ACM.0000000000004920 35947473

[2] Kalet A, Zabar S, Szyld D, et al. A simulated “night-on call” to assess and address the readiness-for-internship of transitioning medical students. Adv Simul. 2017. 10.1186/s41077-017-0046-1. 10.1186/s41077-017-0046-1 PMC5806245 29450014

[3] Kochmar E, Vu DD, Belfer R, Gupta V, Serban IV, Pineau J. Automated personalized feedback improves learning gains in an intelligent tutoring system. Artif Intell Educ. 2020. 10.1007/978-3-030-52240-7_26.

[4] Yamamoto A, Koda M, Ogawa H, Miyoshi T, Maeda Y, Otsuka F, Ino H. Enhancing medical interview skills through AI-simulated patient interactions: nonrandomized controlled trial. JMIR Med Educ. 2024. 10.2196/58753. 10.2196/58753 PMC11459107 39312284

